# The Right to Protest During a Pandemic: Using Public Health Ethics to Bridge the Divide Between Public Health Goals and Human Rights

**DOI:** 10.1007/s11673-023-10235-w

**Published:** 2023-03-16

**Authors:** Stephanie L. Wood

**Affiliations:** 1 University of New South Wales, High St, Kensington, New South Wales, 2052 Australia; 2 Alfred Health, 55 Commercial Rd, Melbourne, Victoria 3004 Australia

**Keywords:** Public health, Public health ethics, Human rights, COVID-19, Protest rights, Health policy, Legislation

## Abstract

Public protest continued to represent a prominent form of social activism in democratic societies during the COVID-19 pandemic. In Australia, a lack of specific legislation articulating protest rights has meant that, in the context of pandemic restrictions, such events have been treated as illegal mass gatherings. Numerous large protests in major cities have, indeed, stirred significant public debate regarding rights of assembly during COVID-19 outbreaks. The ethics of infringing on protest rights continues to be controversial, with opinion divided as to whether public health goals or human rights should take precedence. This paper applies public health ethical theory to an in-depth analysis of arguments on both sides of the debate. Using the Nuffield Council on Bioethics framework as a backdrop, proportionality and necessity of restrictions are understood as key concepts that are common to both public health and human rights perspectives. The analysis presented here finds a middle-ground between the prevailing arguments on opposing sides and is further able to rationalize the use of protest itself as an important element of a mature public health ethics response to restrictive policy. Thus, this paper aims to influence public health policy and legislation regarding protest rights during public health emergencies.

## Introduction

The COVID-19 pandemic has tested the lengths to which democratic societies are willing to act to prioritize public health and, to this extent, the boundaries of legislative emergency health powers. Restrictive policy, including lockdowns and stay-at-home orders, widely applied during the pandemic, represent breaches of human rights sanctioned for the purpose of meeting public health goals. Debate continues as to what human rights violations are appropriate or acceptable in the context of an active pandemic, with the right to protest one particularly contentious issue.

In this paper, the ethics of the right to protest in the face of public health restrictions on mass gatherings is explored in the context of the COVID-19 pandemic in Australia. Arguments aiming to justify bans on protests emphasize the importance of public health goals; on the other hand, those in favour of unequivocal protest rights take a human rights-based approach, asserting that such activism is an essential part of democratic society. Central to the ethical reasoning of both perspectives are two key concepts: proportionality and necessity. However, the applications of these constructs in the prevailing discourse on the issue of protest rights during a pandemic have been at odds with one another.

This pitting of public health and human rights against each other as ideologically contrary is not constructive to society, nor useful in managing public policy during a pandemic. Instead of framing these as contradictory perspectives, the ultimate argument made here is for balance between the public health and human rights-based approaches through a more nuanced interpretation of public health ethics. Weighing opposing interpretations of necessity and proportionality, and drawing on more progressive public health ethics theory, this paper articulates a more responsive and adaptive ethical response that bridges this divide, respecting both public health goals and the right to protest.

## The Democratic Right to Protest in Australia

Article 21 of the International Covenant on Civil and Political Rights, of which Australia has been a signatory since 1980, articulates the right of peaceful assembly (Department of Foreign Affairs and Trade 1998). One of the few exceptions to the unrestricted nature of this democratic right is for the purpose of protecting public health (Department of Foreign Affairs and Trade 1998). Locally, Australia has no human rights act at the national level and thus has not federally legislated the right to protest (Martin 2021; Amnesty International 2021a). State and territory legislation is variable; Victoria, the Australian Capital Territory (ACT), and Queensland have implemented some provisions in their human rights acts, whilst the New South Wales (NSW) equivalent does not specifically call out assembly rights (Martin 2021; Human Rights Law Centre 2020b). Although not explicitly articulated in the Constitution, legal challenges have previously upheld protest rights based on implied freedoms (Anderson 2021; Martin 2021; O’Sullivan 2020a).

During the COVID-19 pandemic, numerous protests have taken place in Australia. Prominent underlying issues have included the Black Lives Matter (BLM) movement, treatment of refugees in detention, and opposition to pandemic restrictions themselves, with most social activism taking place in the capitals of New South Wales and Victoria (O’Sullivan 2020b). Stay-at-home orders did not list protests as an essential activity nor include them in lists of exempted activities; hence, these activities fell to the remit of broader bans on mass gatherings (Anderson 2021; Legal Observers NSW 2021; O’Sullivan 2020b).

## The Perspective of Public Health Ethics—The Nuffield Framework and Coercive Public Health Restrictions

The Nuffield Council on Bioethics (NCB) intervention ladder comprises an eight-step hierarchy of government actions that can be taken to address a public health issue, underpinned by a stewardship model articulating the goals and constraints of interventions (Nuffield Council on Bioethics 2007). Escalating rungs on the ladder represent increasing levels of intrusion on civil liberties, which in turn require increasing justification of public health actions (Nuffield Council on Bioethics 2007). Due to its core emphasis on the need for public health to balance interventions against civil liberties, the Nuffield framework is appropriate for evaluating the ban on protests in Australia under COVID-19 restrictions.

The removal of the right to protest represents the highest rung on the Nuffield ladder—the elimination of choice (Nuffield Council on Bioethics 2007). In the context of the stewardship model, these restrictions represent constraints that are particularly coercive, intrusive, and lacking in individual consent, with goals requiring strong justification (Nuffield Council on Bioethics 2007). An element of the Nuffield framework that is useful in judging the appropriateness of such coercive public health policy as the ban on protests, particularly in light of the opposing arguments discussed below, is **proportionality** (Nuffield Council on Bioethics 2007). The Nuffield framework articulates proportionality as a three-pronged component of a precautionary approach, comprising a balancing test, suitability test, and necessity test (Nuffield Council on Bioethics 2007).

The **balancing test** seeks to confirm that public health goals are important enough that the intervention is appropriate for consideration (Nuffield Council on Bioethics 2007). To this extent, NCB views the threat of a global infectious disease pandemic as both an urgent and serious problem in which policymakers may be compelled to act quickly in restricting the rights of people across populations (Nuffield Council on Bioethics 2007). Such justification invokes the underpinnings in their stewardship model of Mill’s harm principle. Mill’s harm principle holds that the only justification for exercising power over someone against their will is if this will prevent harm to others (Nuffield Council on Bioethics 2007). In applying this principle, NCB places emphasis on the protection of others, specifically accepting state interventions that infringe on individual liberty only under this condition (Nuffield Council on Bioethics 2007). Thus, restrictions to the right to assembly pass the balancing test.

The **suitability test** judges whether an intervention will achieve the desired outcome (Nuffield Council on Bioethics 2007). In this regard, ethical reasoning points to normative assumptions about the spread of airborne viruses in mass gatherings (Mujica et al. 2020; Walsh et al. 2021; Towell and Cowie 2020) and social distancing as a widely accepted mechanism of pandemic control (Nuffield Council on Bioethics 2007). There are some unique characteristics of protests that add to this argument. Shouting and chanting increase airborne spread of virus particles (Towell and Cowie 2020), as does the use of tear gas and pepper spray for crowd control, since this leads to protesters removing their masks, sneezing, and coughing (Eisenman et al. 2021). In this context, large protests have the potential to turn into “super-spreader events” (Eisenman et al. 2021). Thus, applying the harm principle, a ban on protests achieves the aim of public protection in more ways than one.

The last element of proportionality in the Nuffield framework is the **necessity test**, which compels policymakers to choose the intervention that is least intrusive and restrictive to civil liberty (Nuffield Council on Bioethics 2007). Here, NCB considers social distancing and ban on mass gatherings to be inherently less restrictive to liberty than other public health measures during a pandemic, since individuals can continue to live their life otherwise unimpeded (Nuffield Council on Bioethics 2007). Choosing the least intrusive and restrictive measures also demands a consideration of alternatives. In this regard, public health proponents have argued that alternative means of activism are available through online and social media, and that protests could be delayed until a more appropriate time (Barclay et al. 2020; Martin 2021; O’Sullivan 2020a; Towell and Cowie 2020).

This argument is even stronger where protests directly oppose public health goals (e.g., anti-mask, anti-vaccination), are based on misinformation or conspiracy theories, are violent, or where politically-motivated leaders encourage dissent (Bongiorno 2021; Dyer 2020; Taylor 2021; Day and Carlson 2021; Breakey 2021). Moreover, the pandemic has seen increases in violent activism and extremism (Taylor 2021; Day and Carlson 2021; Premier of Victoria 2021), involving actions that would not be tolerated even in non-pandemic times, given the existence of other laws governing the ways in which protest can be held (Anderson 2021; Attorney-General’s Department n.d.; Breakey 2021). Here, NCB would again invoke the harm principle, maintaining that, ultimately, violent and antisocial protest lies at odds with the protection of others; as such, the banning of this behaviour would not represent a restriction of liberty under the Nuffield framework (Nuffield Council on Bioethics 2007).

## The Perspective of Human Rights—Proportionality and Necessity

In considering the role of human rights in this debate, it is pertinent to again note the lack of relevant legislation in Australia. As compared with the Nuffield framework for public health, there is no comprehensive, formal human rights-based framework that can be drawn upon to rationalize the safeguarding of the freedom to protest (Victorian Equal Opportunity and Human Rights Commission n.d.). The right to protest is nonetheless fundamental to democracy from a human rights perspective, addressing inequity and improvement of country (Human Rights Law Centre 2020a). Historically, legislation on important issues has been propelled by collective action through protests (Amnesty International 2021b, Human Rights Law Centre 2020a). Regarding the need for in-person events, the nature of protests to cause disruption is considered key to their effectiveness in prompting government action (Ricketts 2021; O’Sullivan 2020b). Banning in-person protest thus raises concerns about the censuring of free speech and broader arguments that Australia tends to criminalize dissent (Barclay et al. 2020; Martin 2021). Given these considerations, human rights advocates contend that pandemic restrictions on protests should be both **proportionate** and **necessary** (Amnesty International 2021a; O’Sullivan 2020a). Herein, there are significant tensions in the interpretation of these concepts on the two sides of the debate.

Deliberation on the proportionality of protest bans during COVID-19 has primarily centred on a balancing of restrictions against infection risks (Langton 2020; O’Sullivan 2020b). However, lawmakers have often failed to mount convincing arguments demonstrating that these infection risks outweigh contemporaneous human rights issues. For example, Aboriginal and Torres Strait Islander communities fared relatively well for most of the pandemic, with only 153 cases and no deaths in the first year of the pandemic (to late April 2021) (Australian Institute of Health and Welfare 2021), and the first COVID-19 fatality in an Aboriginal and Torres Strait Islander person occurring in August 2021 (Griffiths 2021). This is in contrast with the significant inequities and human rights issues facing this population, notably, the high rate of arrest and incarceration of First Nations Australians and fifteen Aboriginal and Torres Strait Islander deaths in policy/prison custody in 2020–2021 (sixteen in 2019-2020) (Doherty 2021), including numerous, high-profile cases calling into question the treatment of First Nations peoples in custody and circumstances of deaths (Archibald-Binge 2021; Allam 2020, 2021; Prosser 2020; Dillon 2021; Fox Koob 2021).

Despite COVID-19 presenting an arguably lower risk to First Nations people’s health at the time, BLM protests held in 2020, highlighting the ongoing and active issue of violence against Aboriginal and Torres Strait Islander peoples in custody, were condemned as inappropriate in the context of the public health situation (Langton 2020; Anthony et al., 2020). On the other side of this coin were protests regarding refugee health, with activists fighting for better living conditions for the purpose of greater protection from COVID-19 (O’Sullivan 2020b). In this case, even though their cause seemingly aligned with greater public health goals, these protests were also disallowed (O’Sullivan 2020b).

Similar to the Nuffield framework, human rights arguments around proportionality ask whether there exist appropriate alternative measures to protest restrictions (O’Sullivan 2020a). This is where the similarity ceases, though; whilst NCB can justify restriction of such liberties against the pandemic risk, human rights arguments set a higher bar for proportionality. Alternatives to complete bans have been suggested, such as “COVID-safe” (Legal Observers NSW, 2021) protests, with limits on crowd size and mandates for outdoor settings, mask use, and vaccination (Legal Observers NSW, 2021; Corpuz 2021; Valentine et al. 2020; Human Rights Law Centre 2020a). Such alternatives are supported by evidence suggesting small protests may have only a minor impact on case numbers (Neyman and Dalsey 2021). However, even in the setting of COVID-safe protests, police actions have raised concerns regarding proportionality (Amnesty International 2021a; Legal Observers 2021).

The police handling of COVID-safe protests in both NSW and Victoria has drawn criticism from human rights advocates who have argued the injustice of fines, arrests, and photographing of approved legal observers, especially given the contrasting, more permissive treatment of other outdoor events during the pandemic (Legal Observers 2021; Human Rights Law Centre 2020a; O’Sullivan 2020b). The use of force and pepper spray on NSW protestors was considered both disproportionate and unnecessary and further compromised the ability of demonstrators to behave in a COVID-safe manner, calling into question whether restrictions truly represented legitimate pandemic control measures (Amnesty International 2021a).

The concept of necessity can also be used to compare the urgency of goals of public health with those of protest action. Countering the argument that restrictions are necessary for pandemic protection, human rights advocates emphasize the time-critical need to address ongoing social injustices through protest. Locally, protests regarding the BLM movement and refugee rights have sought to add much needed momentum to addressing these human rights abuses (O’Sullivan 2020b; SBS News 2020). Drawing criticism on the global stage, both the disadvantage experienced by Aboriginal and Torres Strait Islander peoples and Australian policy regarding offshore processing and detention of refugees were highlighted as pressing issues in the Human Rights Watch thirty-first annual World Report (Human Rights Watch 2021).

Internationally, there are similar examples of social justice activism during the pandemic. In Poland, following the 2020 overturning of a legal exception permitting abortions for severe foetal abnormalities, activists took to the streets in protest, notwithstanding the prohibition of public assemblies, with increasing turnout despite escalating COVID-19 case numbers (Makowska et al. 2021; Associated Press 2020). The significance of this issue to women’s health and the public sentiment was clear in 2021 when further mass protests followed the unnecessary death of a woman who was refused a termination (ABC/Wires 2021; Agence France-Presse (AFP)—SBS and Reuters 2021).

Such tensions between competing health issues are poignantly evident in the nurse strikes regarding COVID-19 workplace safety in South Africa in 2020 (Mulaudzi et al. 2021). Nursing staff were facing dire shortages in personal protective equipment, risking their own lives, patient care, and the broader healthcare system (Mulaudzi et al. 2021). However, protests were banned under mass gathering restrictions, limiting the nurses’ ability to strike and testing their professional ethics in breaching public health orders (Mulaudzi et al. 2021). These examples, in highlighting the weight of issues at the centre of protest action, counter the viewpoint that pandemic control is always the highest priority and, further, that activism can be deferred for a later time.

## Bridging the Divide With Public Health Ethics

Evident in the discourse regarding the right to protest is a conceptual gap concerning necessity and proportionality between the human rights-based arguments and public health goals. Herein, a relevant criticism of the Nuffield ladder is that it is “two-dimensional” (Dawson 2016). Indeed, in applying the Nuffield framework to the issue of protest rights, it can be seen as reductive in its treatment of intrusions to the least degree, rather than incorporating a more malleable conceptualization of liberty (Dawson 2016). As Savulescu contends, “lockdown is a sledgehammer of a solution” (Savulescu 2020), which should only be used as a bridging measure whilst establishing plans that allow a more refined consideration of public health goals (Savulescu 2020). To this end, an application of more nuanced concepts in public health ethics has the potential to meaningfully mediate this debate.

The Childress ethical framework of presumptivism, where public health actors must start with the presumption of civil liberty, articulate their goals and then provide a burden of proof, provides a more flexible construct of liberty in the face of restrictive policy (Childress 2014). In contrast to NCB, this model separates necessity from proportionality, allowing for more complex justificatory conditions (Childress 2014). The presumptivist would hold that, even if a ban on mass gatherings is necessary, it can be applied differentially to different liberties (Childress 2014), for example, freedom of assembly for protest as distinct from attendance at sporting or entertainment events. This avoids a pitfall in Nuffield reasoning that the necessity of protest restrictions is justified through its consequences on pandemic control. Instead, public health is constantly compelled to consider alternatives to limiting protest rights, rather than balance abstract notions of liberty and public health goals (Childress 2014).

A more evolved understanding of paternalism furthers the Childress ability to consider restrictions that expressly distinguish between civil liberties. Blanket bans on protests in COVID-19 can be seen as an expression of traditional, nanny-state paternalism (Carter et al. 2015), where the individual right to freedom of assembly is undermined on the grounds of protection from infectious disease. As such, similar to the NCB emphasis on consequentialism, the nanny-state employs a rigid concept of welfare-justification conditions (Carter et al. 2015). Relational paternalism, however, contends that autonomy is a social construct rather than an individualistic one, and therefore that some civil liberties are more vital than others (Carter et al. 2015). Under this type of paternalism, restrictions on mass gatherings would place minor emphasis on more socially trivial freedoms of assembly, giving credence only to those infringements that affect self-determination, self-governance, and self-authorization, such as protest rights (Carter et al. 2015).

Relational paternalism and presumptivism both incorporate notions of voluntary cooperation (Childress 2014), contrasting with NCB, which positions public acceptance as an endpoint of effective policy (Nuffield Council on Bioethics 2007). This highlights another useful ethical concept: social contract (Kerridge et al. 2013). Social contract theory conceives that individuals are both rational and self-interested, leading to collective will that simultaneously values social justice and is individually acceptable (Kerridge et al. 2013; Nuffield Council on Bioethics 2007). Early in the pandemic when the nature of COVID-19 was unclear, nanny-state paternalistic measures were easier to justify and society was more readily galvanized around the defeat of an unknown enemy. However, ongoing use of coercive public health orders in the longer term, despite increasing availability of alternative measures to protect society, has raised questions on the appropriateness of lengthy infringements of civil liberties (Savulescu 2020). Increased protest activity during the pandemic has indeed been associated with psychological reactance to restrictions that are perceived to threaten personal freedoms (Taylor 2021). For their part, human rights arguments hold that such activism is an important means of public policy debate, acting as a check and balance on restrictive measures (O’Sullivan 2020a). In this context, an understanding that society’s acceptance of restrictions is dynamic and evolving would allow policymakers to regularly renegotiate the social contract regarding the proportionality and necessity of restrictions, bridging the divide between public health and human rights.

## Conclusion

Though a temporary ban on protests could have been justified early in the pandemic, protracted restrictions represent a mismatch between public health goals and human rights, with potential negative consequences for social justice, other important health issues, and open public discourse regarding the acceptability of restrictions. As opposed to the prevailing human rights-based arguments in favour of more permissive protest restrictions, this paper argues for the right to protest using public health ethics, recognizing that pandemic control is not an absolute or singular priority, and that, as the landscape of COVID-19 changed and evolved, so should have the restrictions to important democratic rights.

Applying nuanced theoretical frameworks, this perspective represents a more postmodern conceptualization of public health ethics where ethics is created and not implicit (Roberts and Reich 2002) and demonstrates how human rights need not be contradictory to public health goals. Notably, this reasoning is able to argue for the usefulness of protest itself in guiding decisions on pandemic restrictions. Protest, by articulating the societal acceptance of, and demanding burden of proof for, paternalistic public health measures, may be the medium by which restrictions are appropriately deliberated, the social contract is renewed, and civil liberties restored. Going forward, protection of the right to protest in Australia during a pandemic should be formally addressed through federal legislation and human rights acts (Amnesty International 2021b), with explicit protection of this unique form of assembly as vital to democracy and social justice.
